# Atypical Polypoid Adenomyoma Presenting as Abnormal Uterine Bleeding in a Reproductive-Aged Woman With Prior Hysteroscopic Resection: A Case Report

**DOI:** 10.7759/cureus.117806

**Published:** 2026-10-12

**Authors:** Sophia Weldon, Delaney Kaklamanos, Jessica Evans, Wanda I Torres

**Affiliations:** 1 Medicine, Nova Southeastern University Dr. Kiran C. Patel College of Osteopathic Medicine, Clearwater, USA; 2 Obstetrics and Gynecology, Nova Southeastern University Dr. Kiran C. Patel College of Osteopathic Medicine, Clearwater, USA; 3 Obstetrics and Gynecology, Suncoast Women's Care, Trinity, USA

**Keywords:** abnormal uterine bleeding, atypical polypoid adenomyoma, endometrial pathology, hysteroscopic myomectomy, recurrent uterine lesion, reproductive-age woman, submucosal fibroid

## Abstract

A 34-year-old female (G0P0) with a history of two prior hysteroscopies and fibroids presented to an outpatient obstetrician-gynecologic clinic for abnormal uterine bleeding. Ultrasound confirmed the presence of a submucosal fibroid and an abnormally heterogeneous endometrium. Subsequent hysteroscopic myomectomy and polypectomy were performed, with specimens sent for pathology. The pathology report for both lesions was significant for atypical polypoid adenomyoma (APA), a rare benign intrauterine lesion. This case report highlights the importance of further research on this pathology and its possible implications for the future care of patients with this diagnosis.

## Introduction

Atypical polypoid adenomyoma (APA) is a rare, generally benign uterine lesion characterized by architecturally complex atypical endometrial glands embedded within benign fibromuscular stroma [1,2]. In one retrospective study, APA accounted for only 10-25 cases among approximately 3,600 intrauterine lesions evaluated annually, underscoring its rarity [3]. APA most commonly arises in reproductive-aged women and often presents as a polypoid lesion involving the uterine fundus, lower uterine segment, or endocervical canal [4]. Although its pathophysiology is not fully understood, APA has been associated with hyperestrogenic states and risk factors related to unopposed estrogen exposure, including nulliparity, obesity, and infertility.

Despite its benign classification, APA is clinically significant because it can histologically mimic atypical endometrial hyperplasia, endometrial intraepithelial neoplasia, and well-differentiated endometrioid adenocarcinoma [2,3,5,6]. Histologically, APA demonstrates irregular proliferation of endometrial glands with branching and tubular components. The epithelial tissue may be clustered or dispersed, with surrounding smooth muscle stroma. This stroma contains interlaced bundles with mild atypia and occasional mitotic cells [2]. APA may also coexist with these estrogen-associated proliferative lesions [5]. For this reason, accurate diagnosis is essential to avoid both overtreatment in reproductive-aged patients and undertreatment when premalignant or malignant pathology is present [7]. Histologic evaluation remains central to diagnosis, although immunohistochemical markers may provide additional support in challenging cases [3,7].

The most common presenting symptom is abnormal uterine bleeding [3]. Although the exact mechanism is poorly defined, bleeding may result from disruption of normal endometrial architecture by the polypoid lesion, irregular endometrial shedding, and hormonal imbalance related to excess estrogen exposure [3,4]. APA may also be associated with infertility, particularly when the lesion occupies the lower uterine segment or endometrial cavity, where it may distort the uterine cavity or interfere with implantation [5].

Management depends on patient age, reproductive goals, extent of disease, and concern for associated endometrial neoplasia. Definitive treatment is hysterectomy, particularly for peri- or postmenopausal patients or those who have completed childbearing [5]. In reproductive-aged patients desiring fertility preservation, conservative management may include hysteroscopic transcervical resection, polypectomy, and/or endometrial curettage [8]. Lesion recurrence is common and can occur after uterine-preserving treatment [5,8,9]. Therefore, close follow-up with repeat sampling or hysteroscopic evaluation is recommended [9]. This case highlights the importance of recognizing APA as a rare but clinically significant lesion in the evaluation of abnormal uterine bleeding, particularly because of its histologic overlap with premalignant and malignant endometrial pathology.

## Case presentation

A 34-year-old female, gravida zero para zero, presented to an outpatient obstetrician-gynecologic clinic in October 2025 with a three-month history of abnormal uterine bleeding, characterized by heavy menstrual flow requiring hourly pad changes, passage of clots, and intermenstrual spotting. Her menstrual cycles occurred every 21 days and typically lasted seven to 14 days. Her menses were associated with aching and cramping, increased pelvic pressure, and urinary frequency. She was not using any contraceptive method and was unsure about her desire for future fertility.

Gynecologic history included abnormal Pap smears with cervical dysplasia and HPV positivity for the past three years, followed by two colposcopies with no abnormal findings, menorrhagia requiring dilation and curettage (D&C) in 2019 in Peru, and multiple fibroids requiring hysteroscopic myomectomy via complete resection using a MyoSure device in 2021. She reported no menstrual complaints following the hysteroscopic myomectomy. Additional significant history included anemia, a BMI of 27.6 kg/m², moderate caffeine consumption, regular exercise, and a family history of breast cancer in the patient's aunt. The patient currently denied gastrointestinal symptoms, postcoital bleeding, dyspareunia, groin pain, easy bruising or bleeding, and prolonged amenorrhea. Physical examination demonstrated an irregular, enlarged uterus measuring approximately 10 weeks in gestational size.

Transvaginal ultrasonography demonstrated an anteverted uterus with a heterogeneous endometrium measuring 26.53 mm in thickness. A 2.7 x 2.56 x 2.83 cm submucosal lesion was identified and interpreted as a submucosal fibroid (Figures 1-2). The patient subsequently underwent hysteroscopic D&C with myomectomy and polypectomy. Both lesions were resected using the MyoSure device, and the specimens were submitted separately for histopathologic evaluation. Endometrial curettage was also performed, with the resulting tissue submitted for pathologic evaluation. Histopathologic examination of both the presumed polyp and fibroid demonstrated APA, characterized by irregular endometrial glands embedded within smooth muscle stroma (Figures 3-6). Immunohistochemical staining for desmin demonstrated diffuse positivity of the stromal component, supporting smooth muscle differentiation within the lesion (Figures 7-8). Comparison with the pathology report from the patient's 2021 hysteroscopy confirmed identical findings of APA.

**Figure 1:**
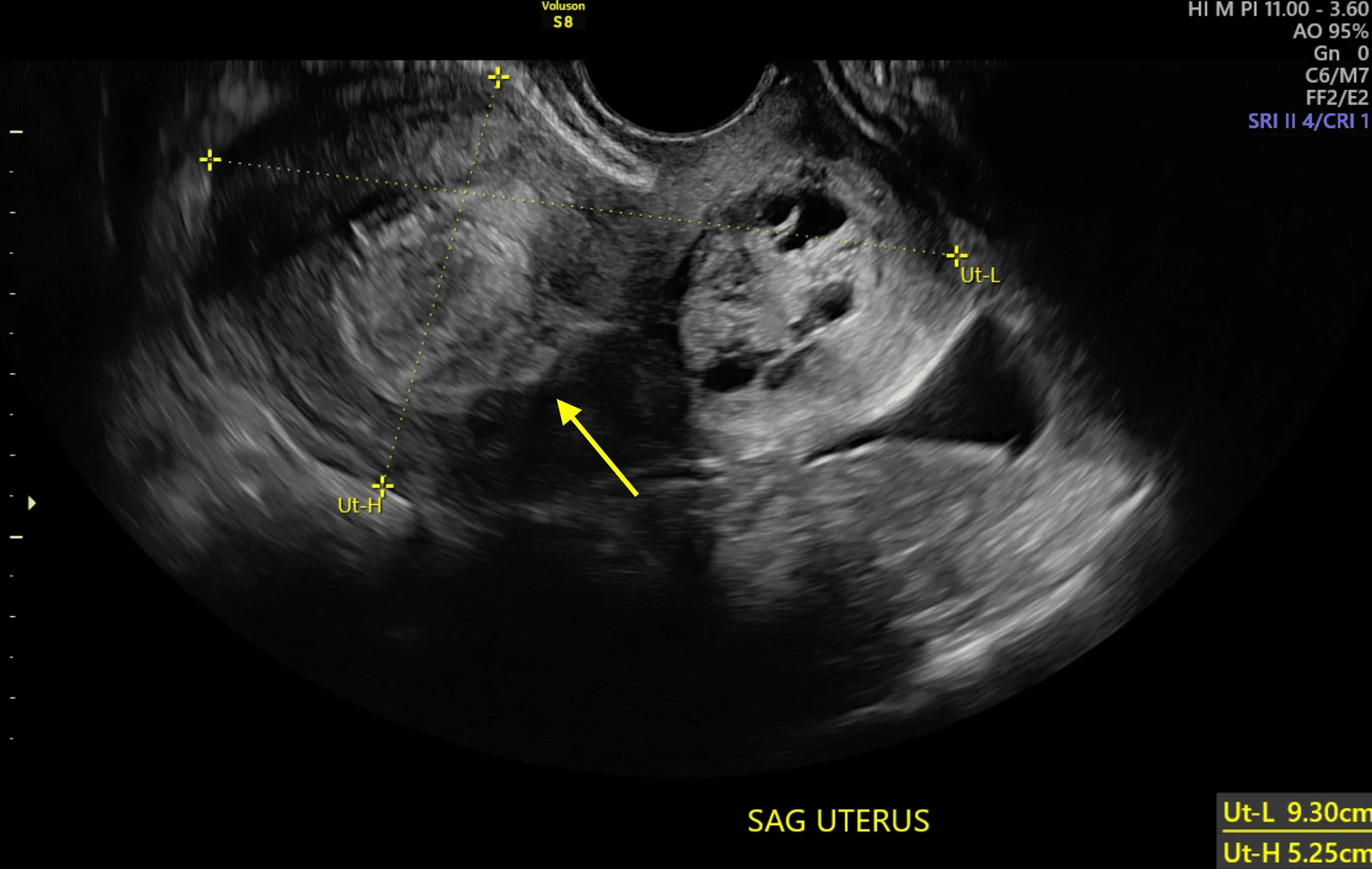
Sagittal transvaginal ultrasound of the endometrium Sagittal transvaginal ultrasound demonstrates a markedly heterogeneous and thickened endometrium. The arrow indicates the lesion, subsequently diagnosed as an atypical polypoid adenomyoma

**Figure 2:**
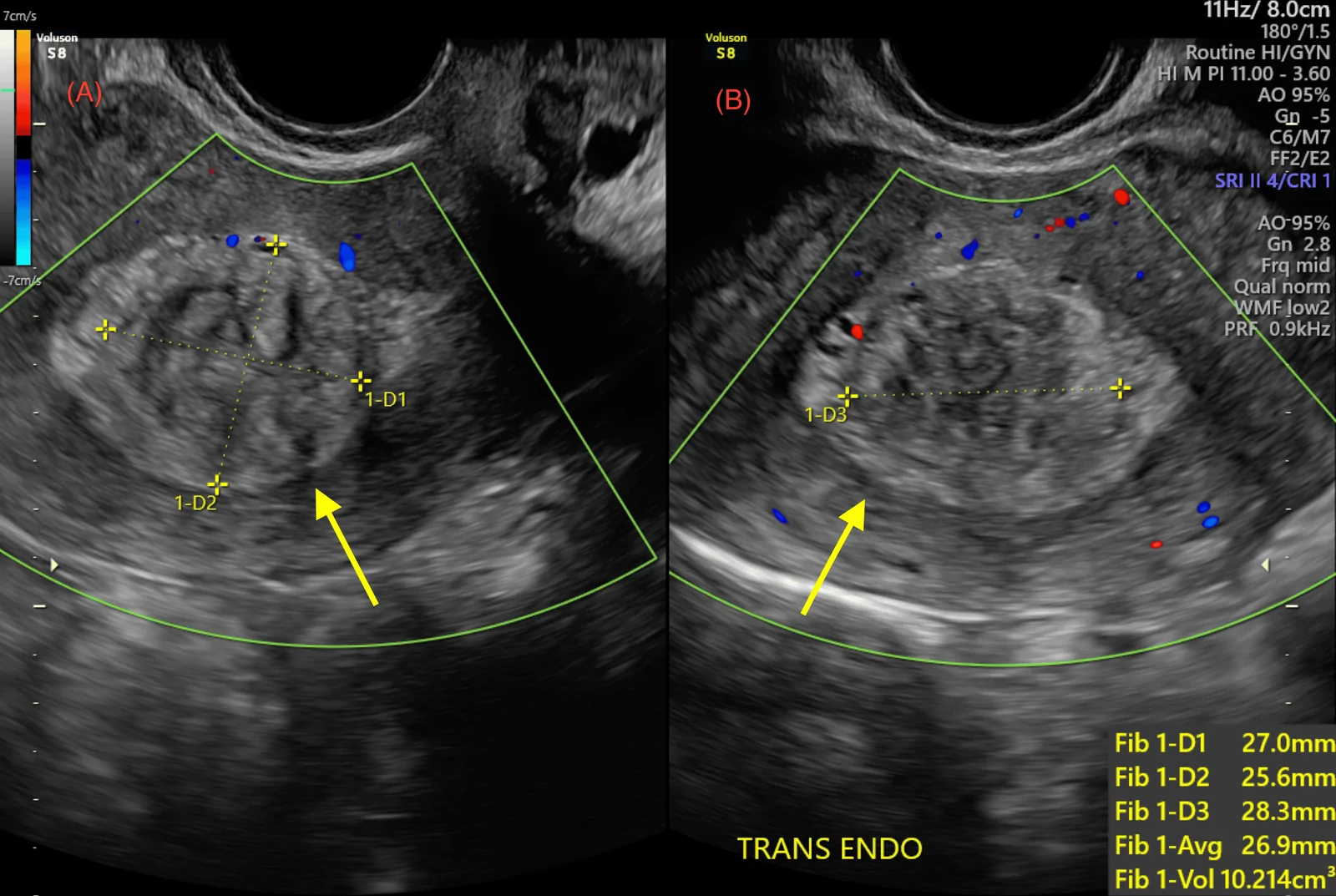
Transverse transvaginal ultrasound with color Doppler of the endometrium (A) Transverse transvaginal grayscale ultrasound demonstrates a heterogeneous endometrial lesion with irregular morphology within the thickened endometrium (arrow). (B) Transverse transvaginal ultrasound with color Doppler demonstrates internal vascularity within the endometrial lesion (arrow)

**Figure 3:**
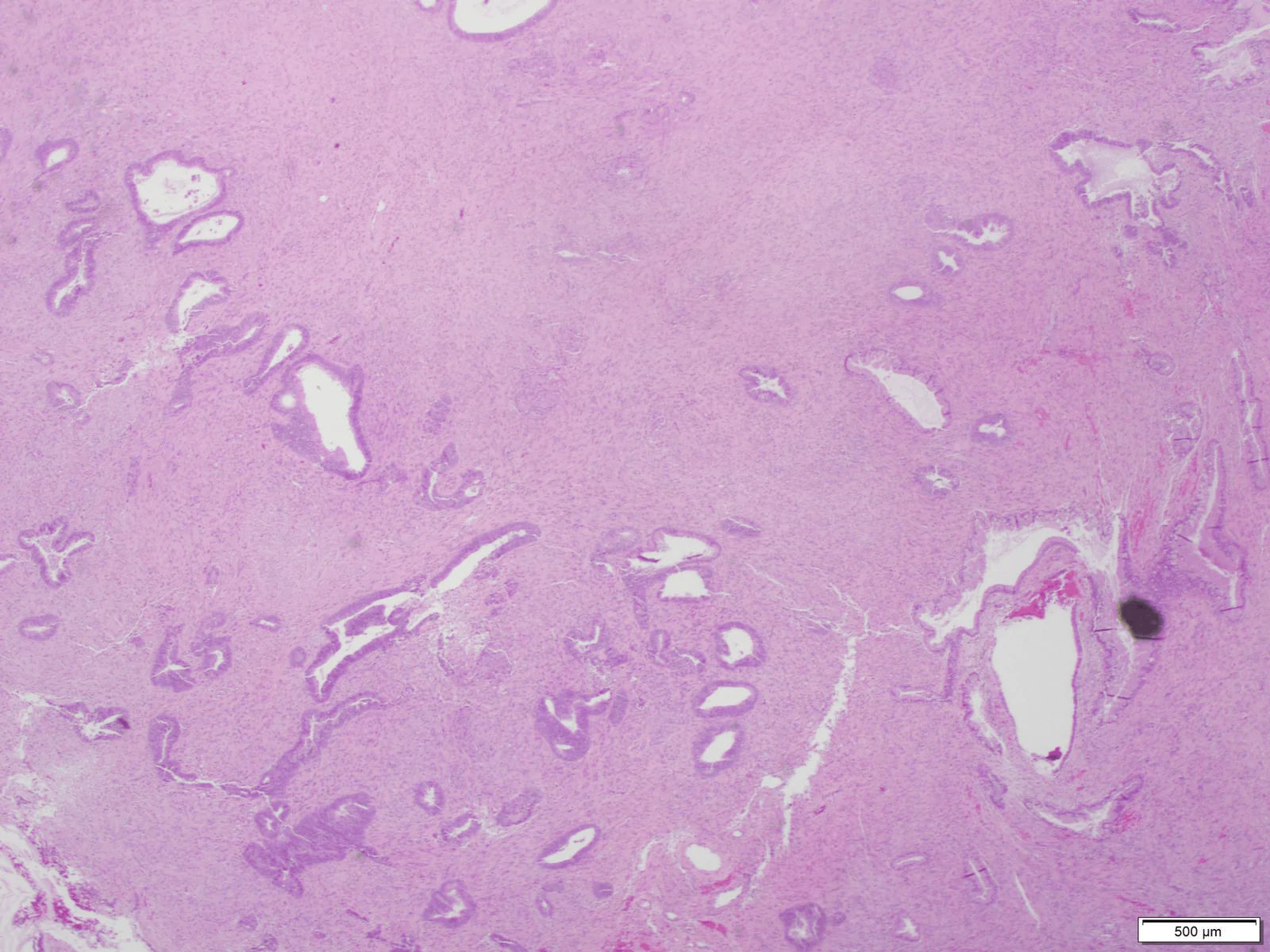
Low-power H&E section - image 1 Low-power hematoxylin and eosin (H&E) section demonstrates a well-circumscribed biphasic lesion composed of irregular endometrial glands embedded within abundant smooth muscle stroma, characteristic of APA H&E: hematoxylin and eosin; APA: atypical polypoid adenomyoma

**Figure 4:**
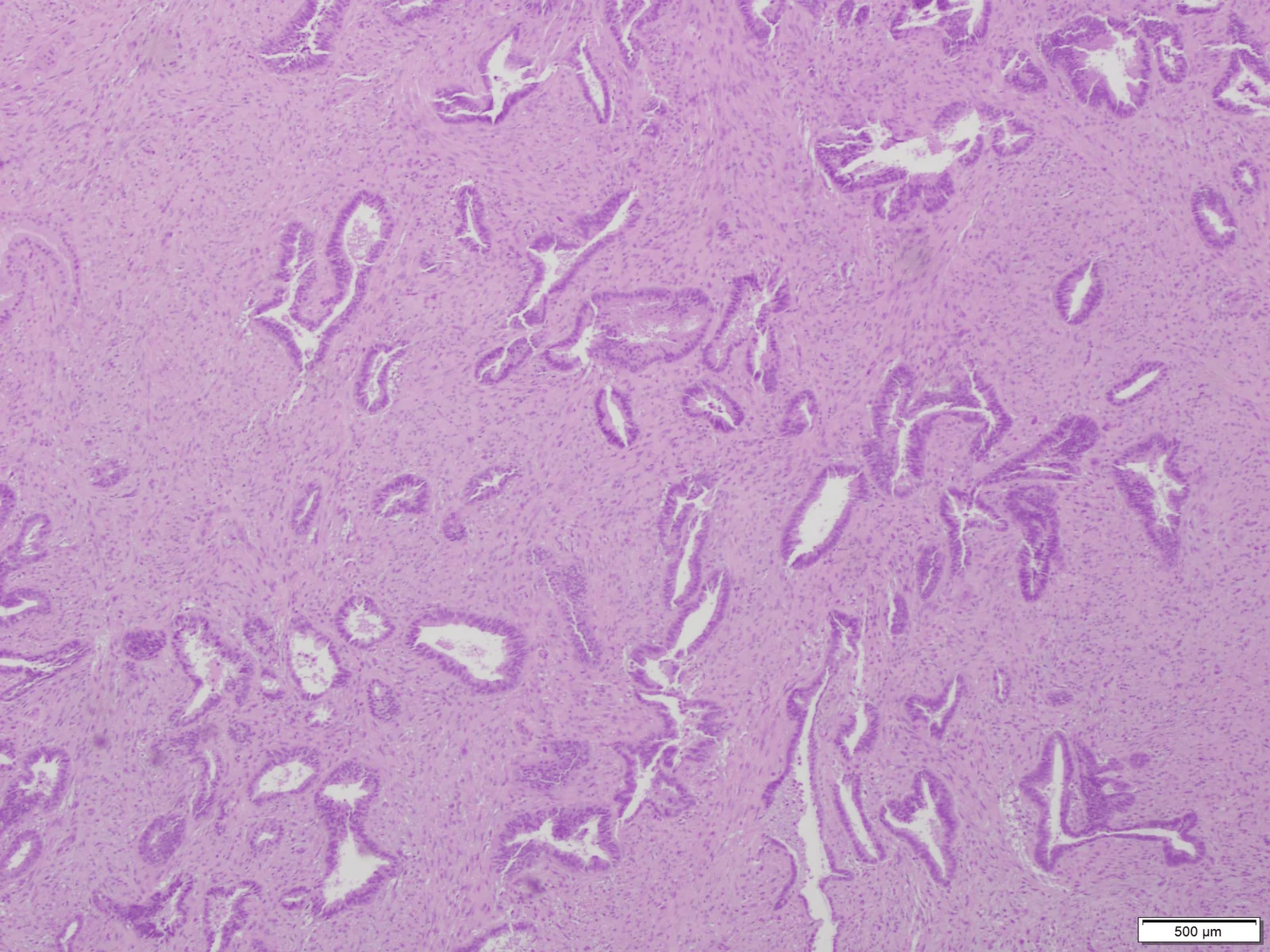
Intermediate-power H&E section Intermediate-power H&E section highlights the complex, irregularly branching endometrial glands surrounded by smooth muscle, illustrating the biphasic architecture of APA H&E: hematoxylin and eosin; APA: atypical polypoid adenomyoma

**Figure 5:**
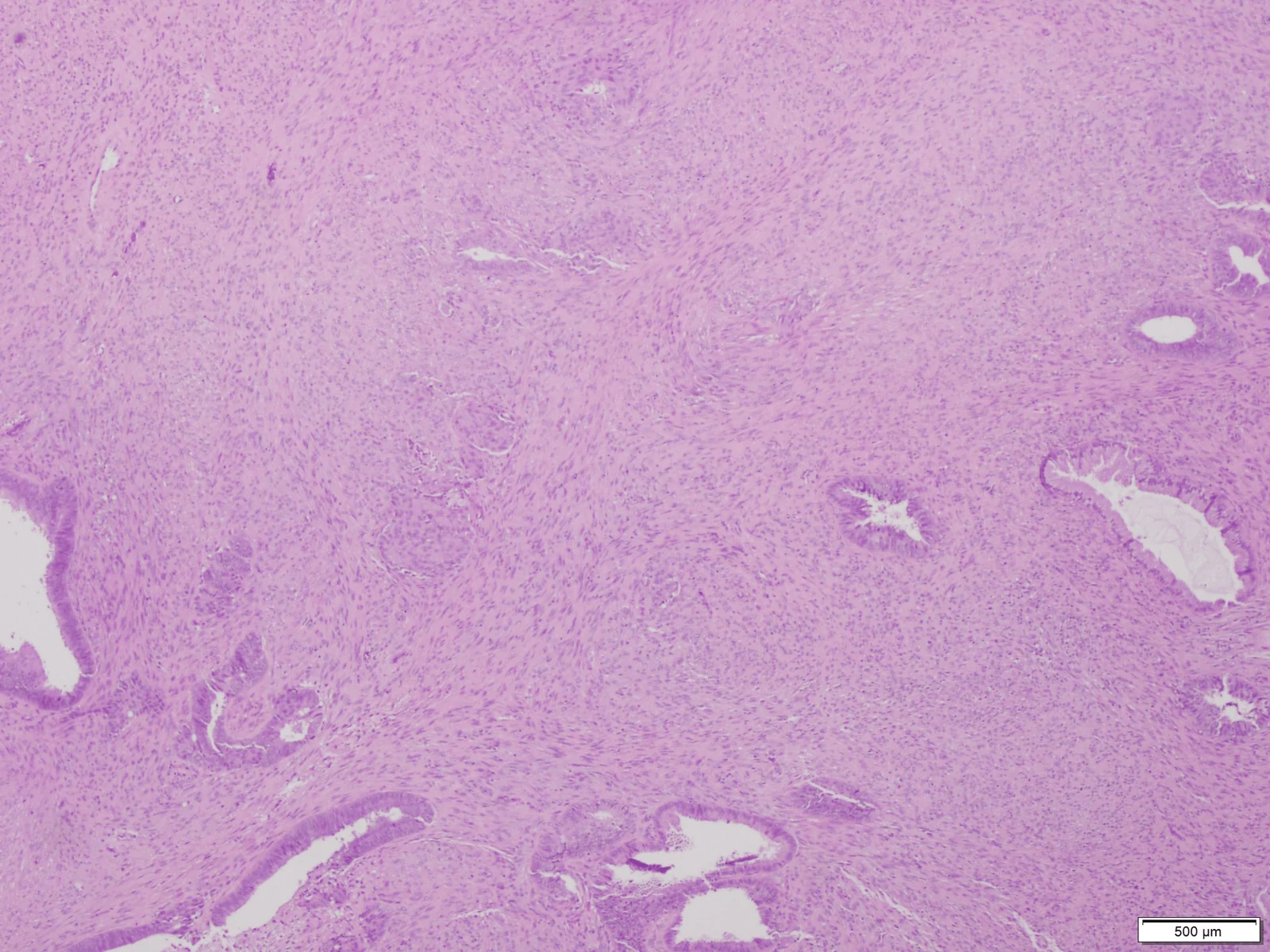
Low-power H&E section - image 2 Low-power H&E demonstrates the swirling smooth muscle stroma surrounding scattered irregular endometrial glands, further illustrating the biphasic glandular and myomatous composition of APA H&E: hematoxylin and eosin; APA: atypical polypoid adenomyoma

**Figure 6:**
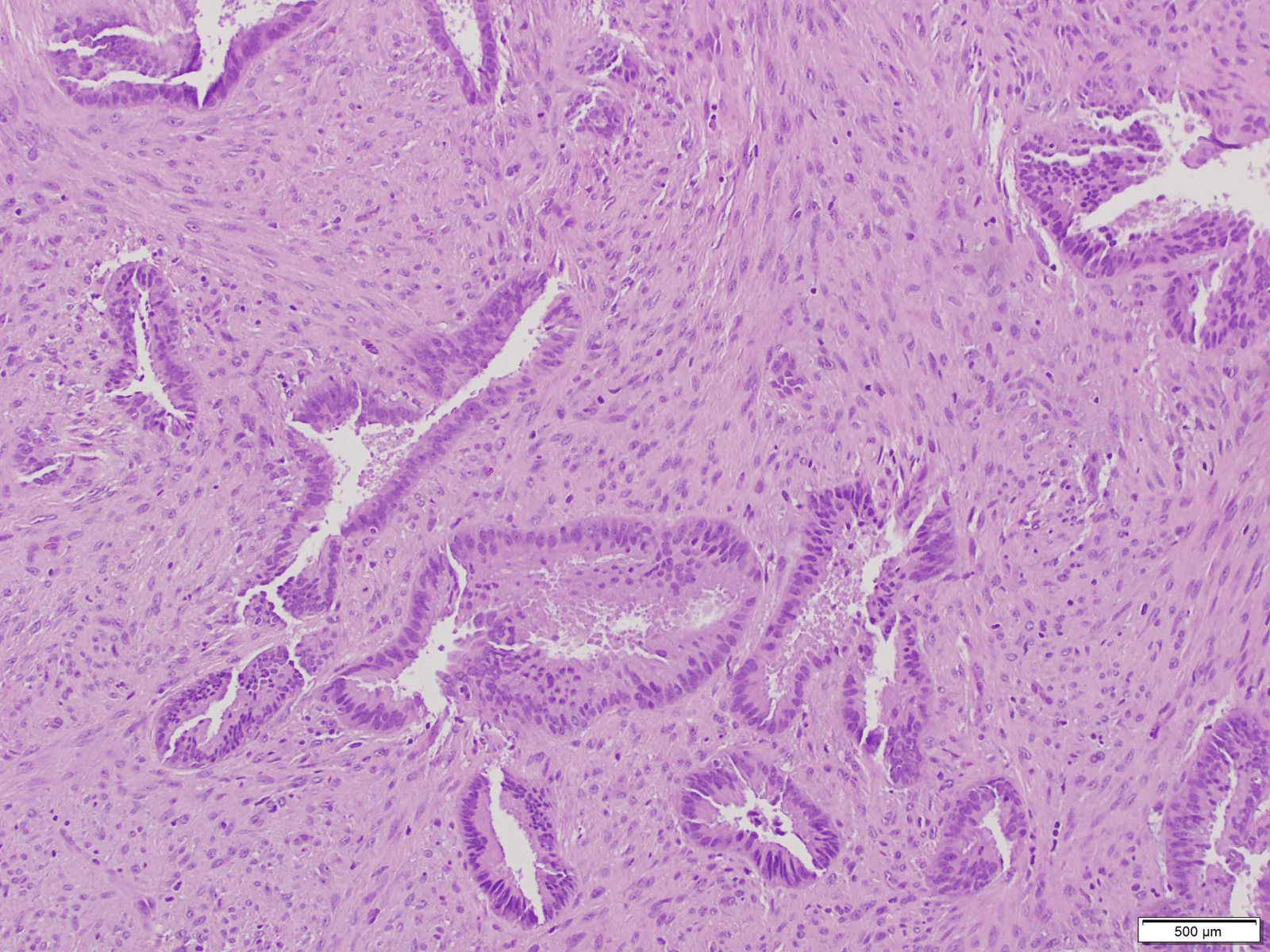
Higher-power H&E section Higher-power H&E section shows architecturally complex endometrial glands lined by mildly atypical endometrial epithelium embedded within a prominent smooth muscle stroma H&E: hematoxylin and eosin; APA: atypical polypoid adenomyoma

**Figure 7:**
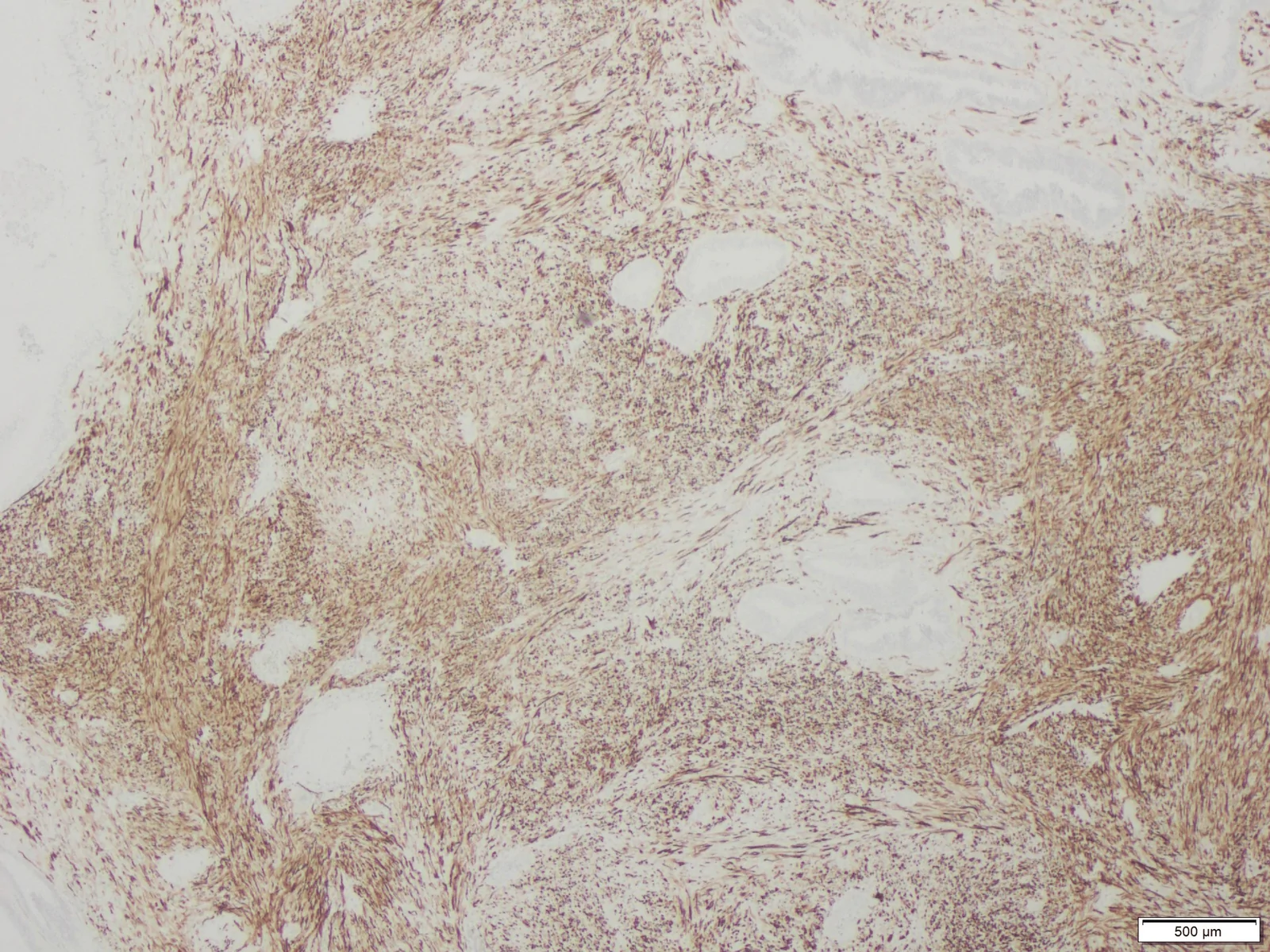
Immunohistochemical staining for desmin The image demonstrates diffuse positivity of the stromal component for smooth muscle markers, supporting smooth muscle differentiation within the lesion

**Figure 8:**
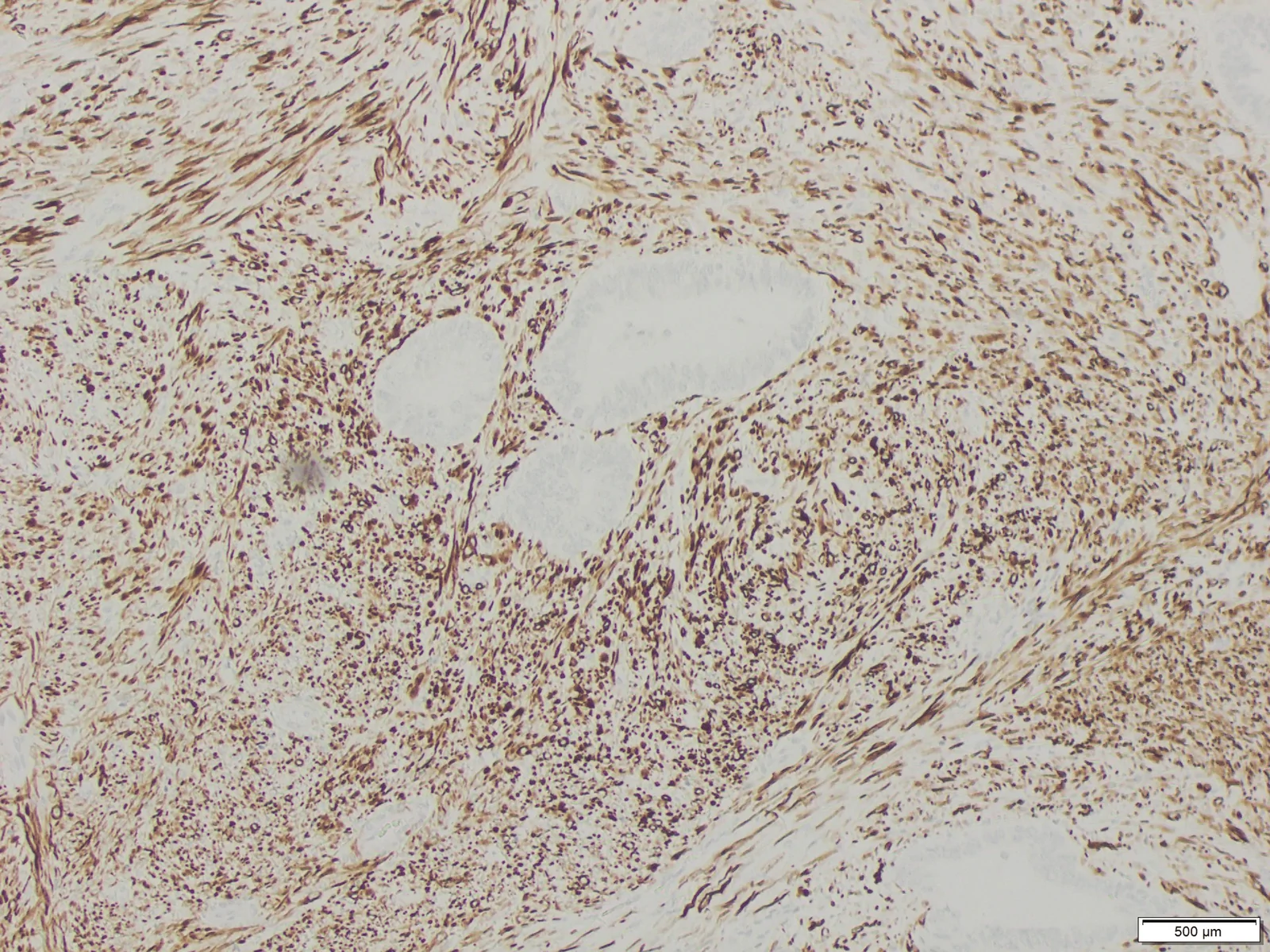
Higher-power immunohistochemical staining for desmin The image shows diffuse staining of the spindle cell stroma surrounding benign endometrial glands, confirming the smooth muscle nature of the stromal component characteristic of APA APA: atypical polypoid adenomyoma

## Discussion

APA remains challenging to diagnose due to both its clinical and radiographic resemblance to more common intrauterine pathologies presenting with similar patient complaints, such as leiomyomas and cervical or endometrial polyps [2,3]. In this case, initial imaging findings suggested a submucosal fibroid with a heterogeneous endometrium, which does not reliably distinguish APA from other benign uterine lesions. The identification of APA in both the presumed fibroid and polyp specimens further highlights the potential for misclassification of these lesions prior to surgical intervention. As seen in this case, histopathologic diagnosis may be difficult, as APA can demonstrate overlapping features with atypical endometrial hyperplasia. Therefore, accurate diagnosis of APA requires careful histopathologic evaluation to distinguish it from other entities [2].

Uterine leiomyomas are benign smooth muscle tumors classified according to the FIGO (International Federation of Gynecology and Obstetrics) subclassification system. FIGO types 0, 1, and 2 are submucosal leiomyomas that distort or extend into the endometrial cavity and commonly contribute to abnormal uterine bleeding [10]. Endometrial polyps are benign overgrowths of endometrial glands and stroma containing characteristic blood vessels and may similarly present with abnormal uterine bleeding [11]. In contrast, APA is a biphasic lesion composed of atypical endometrioid glands embedded within fibromuscular stroma [1,2]. Because APA may appear as a polypoid lesion on transvaginal ultrasound and lacks specific imaging characteristics that reliably distinguish it from these more common entities, histopathologic evaluation is essential when imaging findings are inconclusive. Hysteroscopically directed biopsy or resection allows adequate tissue sampling and identification of the characteristic glandular and stromal components necessary to establish the diagnosis of APA [2].

Likewise, APA may appear similar histologically to atypical endometrial hyperplasia, or both may be present concurrently, leading to difficulty in diagnosis [3,8]. However, endometrial hyperplasia is defined as a premalignant proliferation of crowded endometrial glands and lacks the fibromuscular stroma, which is a defining feature of APA [12]. Because APA may coexist with premalignant or malignant endometrial lesions, adequate sampling of the surrounding endometrium is important to avoid missing concurrent hyperplasia or endometrioid carcinoma and to properly distinguish APA from other lesions [3,8]. Due to this association, APA should be managed with caution, and patients treated conservatively should undergo close follow-up despite its generally benign classification [5].

This case is notable for the identification of APA following prior hysteroscopic resection. The patient’s history of APA on pathology in 2021 raises concern for recurrent disease, although persistent disease due to incomplete resection and development of a new lesion cannot be definitively distinguished. Additionally, this patient demonstrates several risk factors associated with APA, including age, nulliparity, and possible prolonged estrogen exposure, as suggested by the patient’s persistent complaint of menorrhagia and clinical presentation [3,13]. The mechanism underlying APA recurrence is not fully understood, but incomplete removal of the lesion has been proposed as one contributing factor. APA may be difficult to completely excise hysteroscopically, particularly when the lesion extends into the underlying myometrium or when its margins are not readily apparent [5,9].

In this context, the distinction between residual or persistent disease and true recurrence is important. Additionally, APA was identified in both the presumed submucosal fibroid and endometrial polyp specimens in the current presentation, suggesting that thorough evaluation of multiple intrauterine lesions may be particularly important in patients with a prior history of APA. Overall, the repeated or persistent disease in this patient despite prior hysteroscopic resection highlights the need for long-term surveillance and repeat hysteroscopic evaluation in patients who develop persistent or recurrent abnormal uterine bleeding following fertility-sparing treatment [5,8,9].

From a management perspective, this case illustrates the difficult balance between fertility preservation and definitive treatment. While hysterectomy remains curative, it is not the preferred option for patients desiring future fertility [14,15]. Conservative surgical management with hysteroscopic resection combined with close postoperative follow-up and longitudinal surveillance is essential for patients with APA because recurrence rates remain significant among patients managed with fertility-sparing approaches [5,8,9].

Given the low prevalence of APA, standardized management guidelines are not yet adequately established [14]. Greater awareness among providers may improve diagnostic accuracy and contribute to the development of standardized management strategies to ensure appropriate treatment and optimize patient outcomes. In addition, the risk of recurrence and concurrent or subsequent endometrial hyperplasia or carcinoma may require patients treated conservatively for APA to be closely monitored with repeat endometrial sampling or hysteroscopic evaluation, whereas surveillance after definitive hysterectomy is generally guided by the presence or absence of associated premalignant or malignant disease [5,8]. Overall, this case highlights the importance of including APA in the differential diagnosis of abnormal uterine bleeding in premenopausal women, especially in patients with recurrent bleeding despite prior management.

## Conclusions

This case report highlights APA as an uncommon but important cause of abnormal uterine bleeding in reproductive-aged women, including in patients with a history of prior hysteroscopic resection. Its ability to mimic benign entities such as fibroids or endometrial polyps underscores the importance of histopathologic diagnosis. Given its association with endometrial proliferative diseases, close follow-up and individualized management, especially in women desiring fertility, are essential.
